# Carbon Source Reduction Postpones Autumn Leaf Senescence in a Widespread Deciduous Tree

**DOI:** 10.3389/fpls.2022.868860

**Published:** 2022-05-26

**Authors:** Julia Maschler, Jenna Keller, Lalasia Bialic-Murphy, Constantin M. Zohner, Thomas W. Crowther

**Affiliations:** Department of Environmental Systems Science, ETH Zürich, Zurich, Switzerland

**Keywords:** climate change, phenology, autumn leaf senescence, carbon sink limitation, terrestrial carbon sink, carbon cycle, source–sink dynamics, Betula pendula

## Abstract

The growing-season length of temperate and boreal trees has a strong effect on the global carbon cycle. Yet, a poor understanding of the drivers of phenological processes, such as autumn leaf senescence in deciduous trees, limits our capacity to estimate growing-season lengths under climate change. While temperature has been shown to be an important driver of autumn leaf senescence, carbon source–sink dynamics have been proposed as a mechanism that could help explain variation of this important process. According to the carbon sink limitation hypothesis, senescence is regulated by the interplay between plant carbon source and sink dynamics, so that senescence occurs later upon low carbon inputs (source) and earlier upon low carbon demand (sink). Here, we manipulated carbon source–sink dynamics in birch saplings (*Betula pendula*) to test the relevance of carbon sink limitation for autumn leaf senescence and photosynthetic decline in a widespread deciduous tree. Specifically, we conducted a gradient of leaf and bud removal treatments and monitored the effects on autumnal declines in net photosynthesis and the timing of leaf senescence. In line with the carbon sink limitation hypothesis, we observed that leaf removal tended to increase total leaf-level autumn photosynthesis and delayed the timing of senescence. Conversely, we did not observe an effect of bud removal on either photosynthesis or senescence, which was likely caused by the fact that our bud removal treatment did not considerably affect the plant carbon sink. While we cannot fully rule out that the observed effect of leaf removal was influenced by possible treatment-level differences in leaf age or soil resource availability, our results provide support for the hypothesis of carbon sink limitation as a driver of growing-season length and move the scientific field closer to narrowing the uncertainty in climate change predictions.

## Introduction

The photosynthetic uptake of carbon is the biggest flux in the global carbon cycle (Solomon et al., 2007). Over the past decades, established forests have taken up over 25% of the carbon emitted by fossil fuel combustion and land-use change (Canadell et al., 2007; Friedlingstein et al., 2010; Pan et al., 2011). Plant phenology is a major determinant of the inter-annual and seasonal variability in carbon assimilation of temperate and boreal forests (Keenan et al., 2014; Xia et al., 2015; Zohner et al., 2020). As such, accurate predictions of future carbon uptake require an understanding of major phenological processes like spring leaf out or autumn leaf senescence. While the timing of leaf out is mainly driven by temperature (Zohner et al., 2016, 2017), the environmental drivers of autumn leaf senescence are less clear (Gallinat et al., 2015), and predictions of future growing-season length thus remain highly uncertain (Richardson et al., 2012).

Autumn senescence has traditionally been thought to be primarily driven by temperature and daylength (Krol et al., 1995; Rosenthal and Camm, 1997), while additional variation can be explained by factors, such as nutrient (Sigurdsson, 2001) and water statuses (Leuzinger et al., 2005), pathogen infections (Mutz et al., 2021), and air pollution (Gielen et al., 2007). With temperature and daylength as the primary controls of leaf senescence implemented in Earth system models (ESMs; Richardson et al., 2012), global warming is expected to promote earlier leaf out in spring and later autumn leaf senescence, suggesting increased seasonal plant carbon gain (Menzel and Fabian, 1999; Keenan et al., 2014). Yet, there is a growing body of evidence indicating that there are additional drivers of senescence that have been overlooked. For instance, earlier leaf out in spring has shown to be associated with earlier leaf fall in autumn (Fu et al., 2014; Keenan and Richardson, 2015) and an even stronger negative correlation has been found between early-season productivity and autumn senescence dates (Zani et al., 2020). Besides increased water stress resulting from warmer springs (Buermann et al., 2018) or constraints on leaf longevity through accumulation of oxidative damage (Woo et al., 2004), the carbon sink limitation hypothesis offers an explanation for the observed productivity-senescence link (Paul and Foyer, 2001).

According to the carbon sink limitation hypothesis, senescence is regulated by the interplay of the carbon source, that is, organs assimilating carbon, and the carbon sink, that is, net importers of carbon (Paul and Foyer, 2001; Thomas, 2013). With the gradual decline in carbon sink strength over the course of a season, leaves should gradually cease source activity and undergo senescence. Accordingly, larger carbon inputs upon a constant sink size might cause earlier leaf senescence. The concept of carbon sink limitation in trees is supported by a study reporting that individuals that leafed out and senesced earlier had high levels of non-structural carbohydrates (NSC; Fu et al., 2014), which are commonly observed to accumulate in plants upon carbon sink exhaustion (Ainsworth and Long, 2004). There is also evidence from manipulation experiments on herbaceous plants that indicate that the timing of leaf senescence is affected by the balance between the plant carbon source and sink (Guitman et al., 1991; Zhu et al., 2009; Sekhon et al., 2012). If carbon sink exhaustion proves be a driver of autumn phenology in temperate and boreal trees, this suggests that current ESM projections are overestimating the positive effect of higher temperatures, nutrient deposition, or CO_2_ fertilization on plant carbon assimilation under global change.

The manipulation of carbon source–sink dynamics provides means to experimentally test the carbon sink limitation hypothesis. For example, the removal of mature leaves (Guitman et al., 1991) or an elevation of CO_2_ levels (Zhu et al., 2009) increase the potential for carbon uptake. In turn, the carbon sink can be increased by fertilization (Guitman et al., 1991b) or decreased by the prevention of pollination, thereby inhibiting a resource-intensive investment in fruits and seeds (Sekhon et al., 2012). According to the carbon sink limitation hypothesis, we would expect that all measures decreasing the source, or increasing the sink, should postpone autumn leaf senescence, and vice versa. Previous work has provided some initial support for this hypothesis, as source restriction through complete defoliation significantly decreased NSC levels in a study with pines, while sink removal through complete debudding tended to increase carbohydrate accumulation (Li et al., 2002). However, until now, the potential for plant carbon source–sink manipulations to tangibly alter autumn photosynthesis and the timing of leaf senescence remains untested.

Here, we manipulated carbon source–sink dynamics in birch saplings (*Betula pendula*) to test the relevance of carbon sink limitation for autumn leaf senescence in a widespread deciduous tree. Specifically, we conducted a gradient of 25, 50, and 75% leaf and bud removal treatments and evaluated treatment-level differences in leaf-level autumn photosynthesis and the timing of leaf senescence. As a direct evaluation of the carbon sink limitation hypothesis, we tested if leaf removal reduced plant-level carbon assimilation rates, thereby leading to a later satisfaction of the plant’s seasonal carbon demand and later leaf senescence. Similarly, we tested if the removal of buds reduced seasonal carbon demand and thus advanced leaf senescence.

## Materials and Methods

### Plant Material

The experiment was conducted on a terrace of the Swiss Institute of Technology in Zurich, Switzerland, using a total of 81 two-year-old birch saplings (*Betula pendula* L.). On 30 April 2020, we picked up the trees from a local nursery, which had them stored in a cooling room at 4°C since mid-March. On May 1st, we planted the trees in black 5.5 l plastic pots with a heavy, basic-fertilized soil (pH = 6.3–7.8). On June 6th, we fertilized all trees with ~2.15 g NPK fertilizer (DCM ECO-XTRA 1) and ~ 0.55 g of a micronutrient mix (DCM MICRO-MIX). All trees were watered to saturation (i.e., until water started running out from the holes at the bottom of the pots) multiple times per week. On May 1st, as the majority of the trees had already started to leaf out when we obtained them, we classified the trees into one of five leaf-out groups based on the visibility of petioles and the total number and size of unfolding leaves. To correct for differences in leaf-out, trees from each leaf-out group were distributed as evenly as possible among our eight experimental treatments.

### Treatments

To manipulate carbon source–sink dynamics, we created eight treatments with varying leaf and/or bud removal percentages. For the three leaf removal treatments, we removed 25, 50, and 75% of the trees’ leaves. For the three bud removal treatments, we removed 25, 50, and 75% of the trees’ buds. For the 50% leaf + bud removal treatment, we removed 50% of leaves and buds, and for the control treatment, no leaves or buds were removed. A total of ten individuals were assigned to each treatment, and eleven trees made up the control group (*n* = 81).

To maintain leaf removal treatment-level differences over time and account for variation in compensatory growth among treatments, we removed leaves twice (June 11th, August 12th–14th). In the first leaf removal, we haphazardly removed leaves of all sizes. In the second leaf removal, we removed the portion of the natural (control) leaf growth corresponding to the treatment’s specified leaf removal percentage as well as all of the compensatory growth resulting from the treatment (see Supplementary Methods 1). To target the leaves grown since the first leaf removal and thereby decrease differences in average leaf age among treatments, we preferentially removed younger leaves in the second leaf removal. On August 3rd–4th, once the buds had reached a size that allowed for their targeted removal, we removed buds as evenly as possible across their size classes. Both leaf and bud removals were distributed as evenly as possible across the vertical and horizontal axes of the trees. To account for microsite differences on the terrace, we arranged the trees in ten blocks of one tree per treatment each (except for one block being assigned the additional control tree).

### Autumn Photosynthesis Measurements

We measured leaf-level net photosynthesis (μmol m^−2^ s^−1^) on one leaf per tree, using a LI-COR 6800 portable photosynthetic system (LI-6800, Li-Cor, Lincoln, NE). In total, we measured at six intervals between late August and late November 2020, over three to 10 days and usually on consecutive days between 09:00 and 15:00 (Supplementary Table 1). As the LI-COR device can only achieve a temperature differential of 10°C between our 20°C leaf temperature setpoint and the ambient air temperature, we had to move indoors for the complete third to sixth measurement interval. For indoor measurements, plants were moved inside 1 h prior to starting the measurements to ensure sufficient time for trees to acclimate. For our analysis, we assumed that the individual measurements within the same measurement interval were comparable and assigned them all to the same (first) day of the measurement interval. The sample leaf was chosen to be representative of the tree’s leaves in size and color and was located in the middle of the crown vertically. The same leaf was used in consecutive measurements whenever possible, given that it continued to represent a tree’s average leaf; otherwise, a new leaf was chosen. In case leaves were too small to cover the whole LI-COR chamber, we estimated the missing area in scaled photographs using the program Image J Fiji (Schindelin et al., 2012) and converted the photosynthetic rate of small leaves to their full chamber-coverage equivalent. The LI-COR settings were consistent across all measurements (leaf temperature 20°C, light intensity 1,000 μmol m^−2^ s^−1^, see Supplementary Table 2 for a detailed list of settings). Trustworthiness of each photosynthesis measurement was evaluated based on the biological meaningfulness of the additional data in the LI-COR log file (e.g., stomatal conductance cannot be negative), and unreasonable data was removed (see Supplementary Methods 2). Leaf-level net photosynthesis values of trees that had lost all leaves at the end of the season were set to zero. Negative photosynthesis values, which were only obtained in the last two measurement intervals, were also set to zero where biologically meaningful (if the measurement was from the last measurement interval or the tree had lost all its leaves by the next measurement interval); otherwise, the observation was deleted.

We used leaf-level net photosynthesis measurements (after leaf size correction; μmol m^−2^ s^−1^) to calculate two photosynthesis-derived variables: total leaf-level autumn photosynthesis and total relative tree-level carbon gain. Total leaf-level autumn photosynthesis (μmol m^−2^) is defined as the area under the leaf-level net photosynthesis time series curve, which we obtained using the get_auc function from the pmxTools package (Wilkins et al., 2020) in R version 3.5.1 (R Core Team, 2021). We calculated total relative tree-level carbon gain (μmol m^−2^), which is a measure of leaf removal treatment effectiveness corrected for differences in tree size, by multiplying each of the six leaf-level net photosynthesis values per tree with the ratio of the temporarily closest leaf count to the pre-treatment leaf count (see “Autumn Leaf Senescence Measurements at the Whole-Plant Level” for leaf count measurements), followed by calculating the area under the curve using the get_auc function.

### Autumn Leaf Senescence Measurements at the Whole-Plant Level

For calculating autumnal leaf senescence, we measured leaf count and average leaf chlorophyll content using a SPAD-502 Plus leaf chlorophyll meter (Soil Plant Analysis Development, Minolta Camera Co., Ltd., Tokyo, Japan). From mid-August to late November, we measured both parameters 13 times over the course of one to four consecutive days. For our analysis, we assumed that the individual measurements within the same measurement interval were comparable and assigned them all to the same (first) day of the measurement interval. To calculate each tree’s average leaf chlorophyll content, we measured leaf chlorophyll in the upper right corner of all leaves that were big enough to be measured with the SPAD device (first measurement interval) or every third leaf (all other measurement intervals) of a tree and took the average. Later in the season, we sampled chlorophyll of every leaf if the total leaf count of the tree was less than six.

To test the effect of plant organ removal on the timing of whole-plant autumn leaf senescence, we used a tree’s average leaf chlorophyll content and its total leaf count data to calculate an inverted relative SPAD index. Specifically, we first multiplied the tree’s current leaf count $LC_{\mathrm{current}}$ with its average chlorophyll content $\mathrm{avgCh}l_{\mathrm{current}}$ on a specific measurement date. In a second step, we divided the result of the first step by this tree’s maximum result across all measurement dates. Finally, we subtracted 1 from the result of this division and took the absolute value to represent senescence progression on a scale from 0 to 1. Therefore, the inverted relative SPAD index is defined as


(1) $$\begin{array}{l} \mathrm{inverted relative SPAD index}\\ =\mathrm{abs}\left(\frac{LC_{\mathrm{current}}\cdot \mathrm{avgCh}l_{\mathrm{current}}\;}{\max_{i\in N}\left(LC_{i}\cdot \mathrm{avgCh}l_{i}\right)}-1\right)\; \end{array}$$

where i denotes one measurement among all measurements N . While we calculated the inverted relative SPAD index for all 13 leaf count and chlorophyll measurement dates (Supplementary Figure 1), we only used the last nine measurements for modeling leaf senescence over time. We used this September 30th (day of year [DOY] 274) cutoff because it was the date when the inverted relative SPAD index had reached its minimum at the treatment-level. To calculate the DOY of 50% senescence, we used linear interpolation between the DOYs of the two inverted relative SPAD index measurements where the 0.5 threshold was crossed for the last time.

### Data Analysis

To evaluate the effect of our treatments on leaf-level physiology and whole-plant dynamics over time, we fit linear mixed-effects models (LMMs) in R using the lmer function from the lme4 package (Bates et al., 2015). In total, we evaluated the effect of plant organ removal on the following variables: total leaf-level autumn photosynthesis, total relative tree-level carbon gain, leaf senescence over time, and DOY of 50% senescence. For the fully parameterized models of total leaf-level autumn photosynthesis, total relative tree-level carbon gain, and DOY of 50% senescence, we included the *leaf* and *bud removal treatments* and their interaction term as main effects and *block* as a random factor. For the fully parameterized leaf senescence over time model, we included *DOY*, *leaf* and *bud removal treatments*, and interaction terms as main effects and *plant ID* and *block* as random factors.

We checked model assumptions using the residplot function from the predictmeans package (Luo et al., 2021) and—where necessary—transformed the response variable (see Supplementary Methods 3). To test for significant differences among the leaf and bud removal treatments over time and at predetermined time points, we conducted analysis of variance using the ANOVA function in the car package (Fox and Weisberg, 2011). Whenever interactions between the *leaf*- and *bud-removal treatments* and *DOY* were not significant, we dropped them from our model before evaluating treatment effects. We obtained model coefficient estimates using the lmerTest function from the lme4 package and the summary function. The significance level used for all our analyses was *p* = 0.05. For each model, we obtained marginal (variance explained by fixed effects) and conditional *R*^2^s (variance explained by entire model) using the r.squaredGLMM function from the MuMIn package (Bartoń, 2020). Using model-based bootstrapping, we calculated model predictions and approximated 95% confidence intervals, using the bootMer function from the lme4 package. Specifically, we ran 1,000 simulations and obtained the upper and lower limits of the confidence intervals by adding/subtracting the standard error (estimated as the standard deviation of the bootstrap iterations) times 1.96 to/from the predicted value.

### Carbon Source–Sink Manipulation

**Figure 1:**
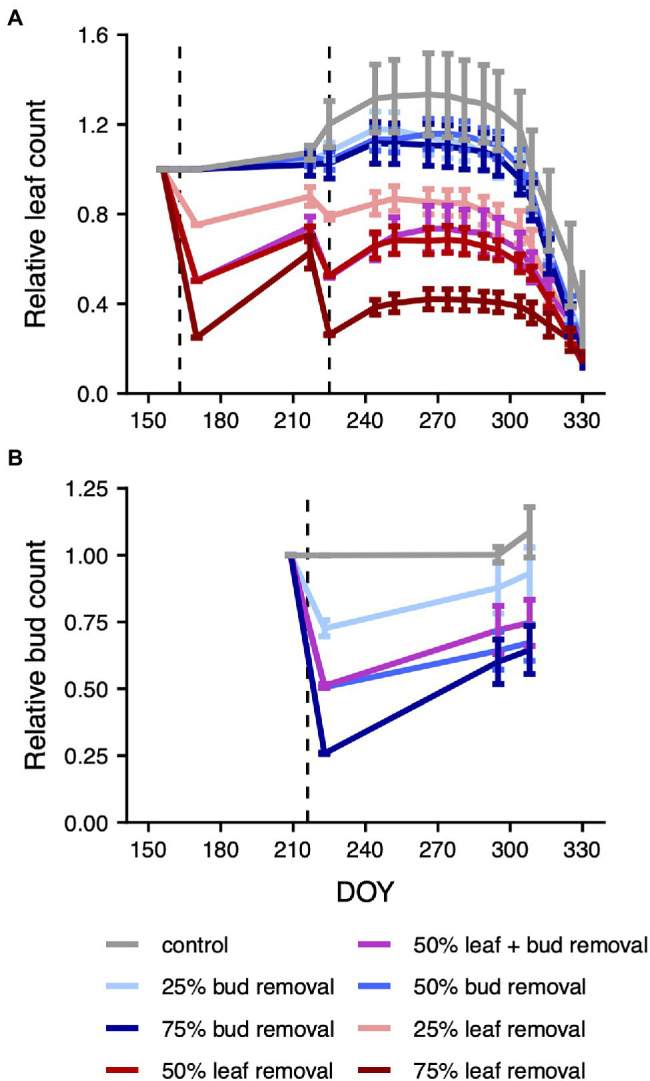
Mean relative leaf and bud counts by treatment over time. The dashed lines mark the DOYs of the leaf **(A)** and bud **(B)** removals. The relative (leaf or bud) count is defined as the ratio of the current count to the count just before the first respective plant organ removal. For the purpose of visualization, we plotted both the first two leaf and bud count measurements 14 days apart from each other, despite them having happened on the same day right before and after the respective plant organ removal. Measurements done over several days were assigned to the first day of the measurement interval. Error bars mark the area of ±1 standard error around the mean. A graph of absolute leaf and bud counts is depicted in Supplementary Figure 2.

Following the first leaf removal, leaf regrowth was particularly pronounced in the leaf removal treatments (Figure 1A, absolute values shown in Supplementary Figure 2A). To offset this imbalance in regrowth rates among treatments, we performed another leaf removal 2 months later. After the second removal, leaf regrowth was more uniform across treatments. We found that leaf removal significantly decreased total relative tree-level carbon gain (*p* = 4.754e-14, degrees of freedom = 1, Supplementary Figures 3, 4; Supplementary Table 3). In contrast, there was no significant effect of bud removal (*p* = 0.757, df = 1). Thus, treatment-level differences in leaf count persisted over the entire season and had the intended effect on the size of the plant carbon source.

Bud removal was conducted shortly before the date of the second leaf removal, and, similar to the situation after the first leaf removal, there was considerable regrowth of buds particularly in the 75% bud removal treatment (Figure 1B, absolute values shown in Supplementary Figure 2B). Treatment-level differences in bud count thus decreased over time after the bud removal, with uncertain consequences for the plant carbon sink.

## Results

**Figure 2:**
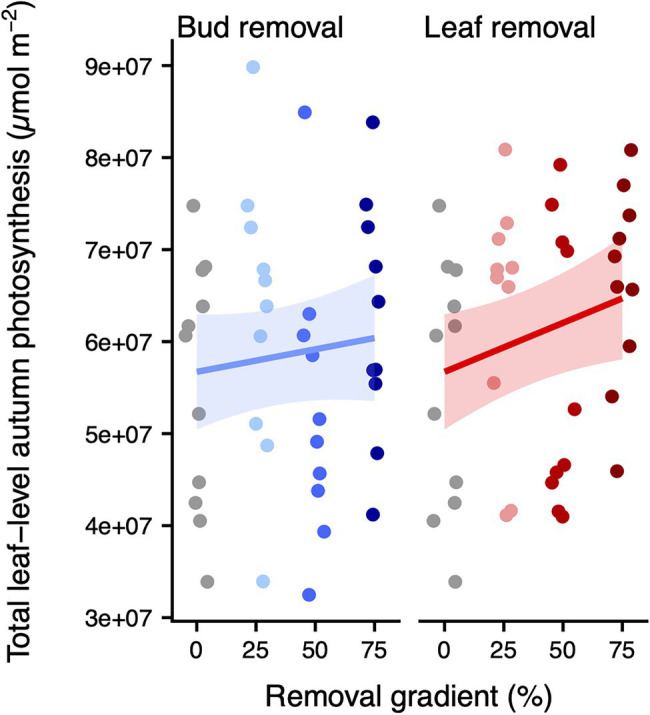
Total leaf-level autumn photosynthesis (μmol m^−2^) across the bud (left panel) or leaf removal gradient (right panel). The colors correspond to the different treatments. For each organ removal, we only display the treatments that have 0% of the other organ removed (i.e., the leaf removal panel does only include treatments where bud removal = 0). We added (back transformed) predicted means and approximate 95% confidence intervals for the model of total leaf-level autumn photosynthesis. Model results are listed in Supplementary Table 4. Model diagnostics are displayed in Supplementary Figure 5.

**Figure 3:**
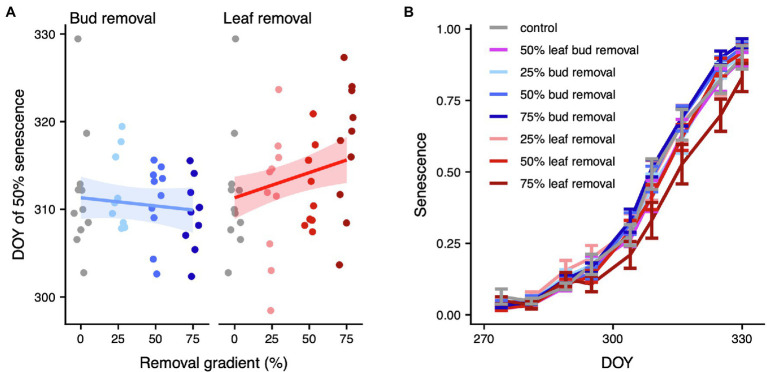
Whole-plant leaf senescence. **(A)** DOY of 50% senescence across the bud (left panel) or leaf removal gradient (right panel). For each organ removal, we only display the treatments that have 0% of the other organ removed (i.e., the leaf removal panel does only include treatments where bud removal = 0). We added predicted means and approximate 95% confidence intervals for the model of DOY of 50% senescence. Model results are listed in Supplementary Table 5. Model diagnostics are in Supplementary Figure 6. **(B)** Whole-plant leaf senescence over time. Error bars mark the area of ±1 standard error around the mean. A graph of the whole time series of inverted relative SPAD index values is illustrated in Supplementary Figure 1. Model results are listed in Supplementary Table 6. Model diagnostics are displayed in Supplementary Figure 7.

We found a marginally significant positive effect of leaf removal on total leaf-level autumn photosynthesis (*p* = 0.054, df = 1), which amounted to ~14% higher total leaf-level autumn photosynthesis upon 75% leaf removal (Figure 2; Supplementary Figure 5; Supplementary Table 4). Also, at the whole-plant level, there were clear differences in leaf senescence across the leaf removal gradient. Specifically, each percent of leaves removed delayed the DOY of 50% senescence by 0.06 ± 0.05 days (*p* = 0.014, df = 1), translating to a delay of 4.31 ± 3.43 days upon a 75% leaf removal (Figure 3A; Supplementary Figure 6; Supplementary Table 5). Consistent with these results, whole-plant leaf senescence over time was sequentially lower across the leaf removal gradient (*p* = 0.020, df = 1; Figure 3B; Supplementary Figure 7; Supplementary Table 6). Conversely, there was no significant change in total leaf-level autumn photosynthesis (*p* = 0.374, df = 1), DOY of 50% senescence (*p* = 0.424, df = 1) or whole-plant leaf senescence (*p* = 0.697, df = 1) across the bud removal gradient.

## Discussion

To improve our understanding of terrestrial carbon fluxes, it is key to understand the factors that determine the length of the active growing season during which trees absorb carbon from the atmosphere. Here, we tested if the manipulation of the carbon source–sink balance affects the timing of autumn senescence in birch saplings. By removing 0, 25, 50, and 75% of leaves from different treatment plants, we generated a carbon source reduction gradient to test if low plant-level carbon assimilation rates would translate into delayed senescence dates. In addition, we removed 0, 25, 50, and 75% of buds on trees, to evaluate whether reduced sink strength might potentially translate to earlier senescence. Our results provide some support for our hypothesis, with trees across the leaf removal gradient tending to have sequentially higher total leaf-level autumn photosynthesis and experiencing delayed whole-plant leaf senescence in autumn, while the bud removal treatment did not show any statistically significant effects. Below, we discuss our results and outline future research avenues to further address the under-researched relationship between source–sink dynamics and growing-season lengths in temperate and boreal forests.

### Effect of Carbon Source -Sink Dynamics on Whole-Plant Leaf Senescence

Following our prediction, we find moderate evidence that our carbon source manipulations increased total leaf-level autumn photosynthesis. Upon a 75% leaf removal, total leaf-level autumn photosynthesis values were ~ 14% higher than in the absence of organ removals. These results fit with findings from another carbon source manipulation experiment at the alpine treeline, where an 80% defoliation in early summer increased autumnal light-saturated net photosynthesis per unit needle area by 7% in larches and 52% in pines (Handa et al., 2005). Therefore, our results are consistent with previous works and lend support to the carbon sink limitation hypothesis by indicating a prolonged physiologically active period upon a reduction of the carbon source. The fact that we did not observe any effect across the bud removal gradient is discussed below in the context of the senescence measurements.

### Effect of Source–Sink Dynamics on Whole-Plant Leaf Senescence

As the degeneration of chloroplasts during leaf senescence leads to a decrease in photosynthetic capacity (Galvagno et al., 2013), we expected a close link between the effect of leaf removal on leaf-level photosynthesis and whole-plant-level leaf senescence. Indeed, the carbon source decrease through a 1% leaf removal delayed the DOY of 50% senescence by 0.06 ± 0.05 days, which amounts to a delay of 4.31 ± 3.43 days upon 75% of leaves removed. This supports the hypothesis that the reduction of the carbon source postpones senescence, which is in line with evidence from studies with herbaceous species. For example, elevated CO_2_ concentrations increased photosynthetic activity in wheat, which accelerated grain carbon sink exhaustion and induced earlier flag leaf senescence (Zhu et al., 2009). In maize, preventing pollination by covering the flowers limited the carbon sink and induced precocious senescence (Sekhon et al., 2012). Recently, a tree study with both experiments and long-term observations showed that an increase in the early- to mid-season carbon source through elevated levels of CO_2_, temperature, and light caused earlier senescence (Zani et al., 2020). In addition, a delay in leaf senescence after a severe summer drought followed by autumn rain in a Central European forest might have been caused by carbon source reduction (Leuzinger et al., 2005). Consistent with these previous studies, we find that leaf removal translates to later senescence, which lends support to the notion that carbon source–sink dynamics at least partially drive senescence patterns not only in herbs but also in trees.

While we found a clear effect of leaf removal on whole-plant leaf senescence and a tendency for higher total leaf-level autumn photosynthesis in defoliated treatments, there are several factors that could have damped the strength of our intended carbon source reduction. Firstly, the late start date of our experiment (small source) and the application of fertilizer (increase in sink) could have led to a relatively large carbon sink in all trees. With wood growth commonly happening until the onset of leaf senescence (Dox et al., 2021), this may have resulted in relatively late occurrence of senescence in general. In this context, a reduction of the source through leaf removal and thereby a slower build-up of NSCs may not have been able to postpone senescence more than a few days relative to the control treatment before other drivers of autumn senescence (e.g., temperature) started to interfere with the effect of carbon source–sink dynamics. Secondly, the effect of our source reduction treatment might have been counterbalanced by unintended reductions of sink strength, for example, stemming from the fact that we had also removed newly emerging leaves during our leaf removal treatments. In addition, sink size might have been reduced through a plant-internal reduction of the carbon sink upon smaller carbon source inputs, for example, due to decreased root growth and thereby also low nutrient uptake. As we monitored neither biomass increments nor soil nutrient concentrations, we cannot evaluate this possibility further. However, it seems most likely that trees subjected to a leaf removal treatment over multiple growing seasons would have a smaller size and thereby also a smaller carbon sink than the control trees (Handa et al., 2005). If this holds within one season, carbon source–sink rebalancing, such as the reduction of the carbon sink upon smaller carbon source inputs, might potentially compensate for initial changes in the two carbon pools and damp the effect sizes for whole-plant leaf senescence.

Similar to the lack of effect of bud removal on photosynthesis measurements, we did not observe any changes in senescence dates across the bud removal gradient. However, it is questionable whether the treatment was effective in removing the trees’ carbon sinks. Due to the small size of the buds in the beginning of the season, we implemented our bud removal late in the growing season (August), meaning that the source–sink relationship was unaffected for the majority of the season. In addition, the considerable regrowth of buds after the removal and the fact that buds are not the primary organs of carbon storage (Landhäusser and Lieffers, 2003; Schädel et al., 2009) indicates that the debudded trees might not have experienced reduced sink activity. As the trees were stored at 4°C until the end of April, bud development was likely also delayed and the resulting carbon sink might have hampered the induction of precocious senescence through debudding (Fracheboud et al., 2009). Furthermore, a removal of buds does not only constitute a carbon sink removal but also a source removal, given that the removed buds cannot open anymore to form photosynthetically active leaves. Accordingly, leaf counts after the second leaf removal in mid-August tended to be lower in the three bud removal treatments relative to the control (Figure 1A). As such, the absence of an effect of the bud removal treatment on senescence dates might have multiple causes and should not necessarily be interpreted as evidence that a reduced carbon sink has no effect on senescence dates. We encourage future experiments to explore alternative means of inducing carbon sink limitation, for example, by reducing the carbon sink through restricted soil resource availability or by increasing the carbon source input through higher light levels.

### Alternative Possible Drivers of the Senescence and Photosynthesis Responses

Besides the carbon sink limitation hypothesis, accumulated resource stress offers an alternative explanation for the observed link between high total relative tree-level carbon gain and earlier senescence in the non-defoliated treatments (Buermann et al., 2018). The higher the productivity of the plant, the more water and nutrients are required over the season. Accordingly, higher total relative tree-level carbon gain and growth rates are associated with a higher risk to experience resource stress (Arendt, 2012), which can in turn induce earlier leaf senescence (Thomas and De Villiers, 1996). We standardized water and nutrient availability across treatments by watering the plants to saturation multiple times per week and providing nutrients in the potting soil and through additional fertilization. This may suggest that the observed treatment differences in senescence and photosynthesis were not driven by variations in nutrient or water availability. However, future research is needed to fully disentangle the effect of soil resource availability and carbon source–sink dynamics on leaf senescence and autumn photosynthesis.

An alternative reason for later autumn leaf senescence and a tendency for higher photosynthesis in the leaf removal treatment could be related to the degree of accumulated oxidative damage, that is, ageing (Woo et al., 2004). In our experiment, there was a positive relationship between the degree of defoliation and leaf regrowth after the first leaf removal. Despite our effort to specifically target recently emerged leaves in the second leaf removal, it is possible that the average leaf age decreased across the leaf removal gradient, which might explain the higher photosynthetic activity and later senescence in leaf removal treatments (Pyung et al., 2007; Zohner et al., 2019). However, as we did not track which leaves emerged before and after the two leaf removals, it is not possible to fully disentangle the effects of leaf age and source–sink dynamics on leaf senescence, and we advise to prevent this caveat in the experimental design of future defoliation experiments by either tracking individual leaf flushing dates or using species like *Fagus sylvatica* that mainly flush at the beginning of the season.

Another possible mechanism underpinning the effect of leaf removal on autumn leaf senescence might have been treatment-level differences in the degree of shading through leaves of the same tree. Self-shading has been reported to decrease carbon gain capacity and thereby reduce the life span of the shaded leaves (Ackerly and Bazzaz, 1995). Accordingly, the higher leaf area index in our non-defoliated treatments might have decreased the lifespan of the shaded leaves. However, the two-year-old birches used in our study did not have a strongly self-shading growth form (e.g., narrow canopy; see Supplementary Figure 8) and they were in direct sun for part of the day. Thus, while self-shading effects among treatments cannot be fully ruled out, these lines of evidence suggest it was not the primary driver of the observed trends in our study.

## Conclusion

Our carbon source–sink manipulation experiment allows us to test the relevance of the carbon sink limitation hypothesis for autumn leaf senescence and photosynthetic decline in deciduous trees. We observed that leaf removal tended to increase total leaf-level autumn photosynthesis and delayed whole-plant leaf senescence, while we did not measure a significant effect across the bud removal gradient. The effect of leaf removal is in line with the carbon sink limitation hypothesis as it suggests that smaller carbon inputs upon a ~ constant sink size delay autumn leaf senescence and the decline in photosynthesis. Yet, we cannot fully rule out that the observed effect of carbon source reduction through leaf removal was influenced by possible treatment-level differences in leaf age or soil resource availability. If carbon sink limitation proves to be a driver of autumn leaf senescence in deciduous trees, this might weaken the strength of the forest carbon sink in future climate change scenarios which project an increase in future growing-season lengths due to the positive effect of higher temperatures, nutrient deposition, or CO_2_ fertilization on plant carbon assimilation under global change.

## Data Availability Statement

The original contributions presented in the study are included in the article/Supplementary Material (Maschler et al., 2022), further inquiries can be directed to the corresponding author.

## Author Contributions

CZ and JM conceived the study design. JK and JM performed the experiment and the analysis and interpretation with the help of LB-M and CZ. JM drafted the article. All authors contributed to the article and approved the submitted version.

## Funding

JM was funded by grants to TC from DOB Ecology. CZ was funded by the Ambizione grant PZ00P3_193646.

## Conflict of Interest

The authors declare that the research was conducted in the absence of any commercial or financial relationships that could be construed as a potential conflict of interest.

## Publisher’s Note

All claims expressed in this article are solely those of the authors and do not necessarily represent those of their affiliated organizations, or those of the publisher, the editors and the reviewers. Any product that may be evaluated in this article, or claim that may be made by its manufacturer, is not guaranteed or endorsed by the publisher.
