# Supplementary material for: Carbon Source Reduction Postpones Autumn Leaf Senescence in a Widespread Deciduous Tree
Source: Front Plant Sci. 2022 May 26;13:868860. doi: 10.3389/fpls.2022.868860 (PMC9199461; doi:10.3389/fpls.2022.868860)
Supplement: Supplementary file 1 [file Data_Sheet_1.PDF]

1. photosynthesis_data.csv

Column	Description
individual	plant ID in the format *Species_number_treatment*
DOY_start_photo	first day of year (DOY) of photosynthesis measurement interval
DOY_photo	DOY of photosynthesis measurement
treatment	/
block	/
GasEx_A_adj	leaf-level net photosynthesis after correction for leaf size
GasEx_A	leaf-level net photosynthesis before correction for leaf size
leaf_out	leaf-out group
leaf_count	(temporarily closest) leaf count
og_leaf_count	original (pre-treatment) leaf count
delta_flow	divergence of flow rate from flow-rate setpoint (500 µmol s^{-1})
delta_tleaf	divergence of leaf temperature from leaf-temperature setpoint (20˚C)

2. senescence_data.csv

Column	Description
individual	plant ID in the format *Species_number_treatment*
DOY_start_sen	first day of year (DOY) of senescence measurement interval
DOY_sen	DOY of senescence measurement
treatment	/
block	/
leaf_count	/
og_leaf_count	original (pre-treatment) leaf count
avg_chl	average tree chlorophyll content
