# Supplementary material for: Carbon Source Reduction Postpones Autumn Leaf Senescence in a Widespread Deciduous Tree
Source: Front Plant Sci. 2022 May 26;13:868860. doi: 10.3389/fpls.2022.868860 (PMC9199461; doi:10.3389/fpls.2022.868860)
Supplement: Supplementary file 4 [file Data_Sheet_4.pdf]

Supplementary Material

Supplementary Figures

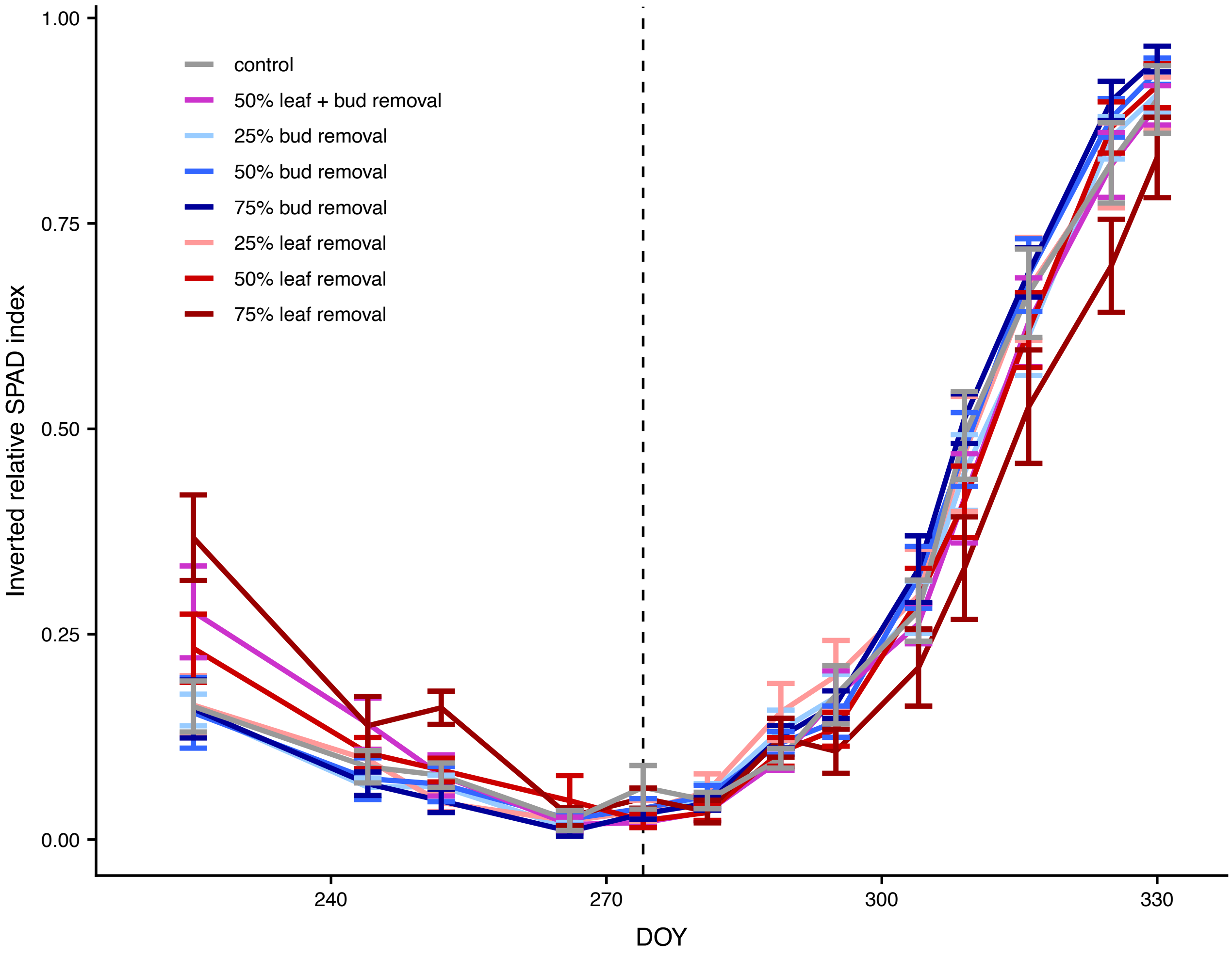

Supplementary Figure 1. Mean inverted relative SPAD index by treatment over time. The dashed line marks the date when the treatment average of the inverted relative SPAD index had reached its minimum in all treatments (DOY 274 = September 30th). The data from the dashed line onwards was used to create the model of whole-plant senescence over time (Figure 3 B, Supplementary Table 6). Error bars mark the area of ± 1 standard error around the mean.

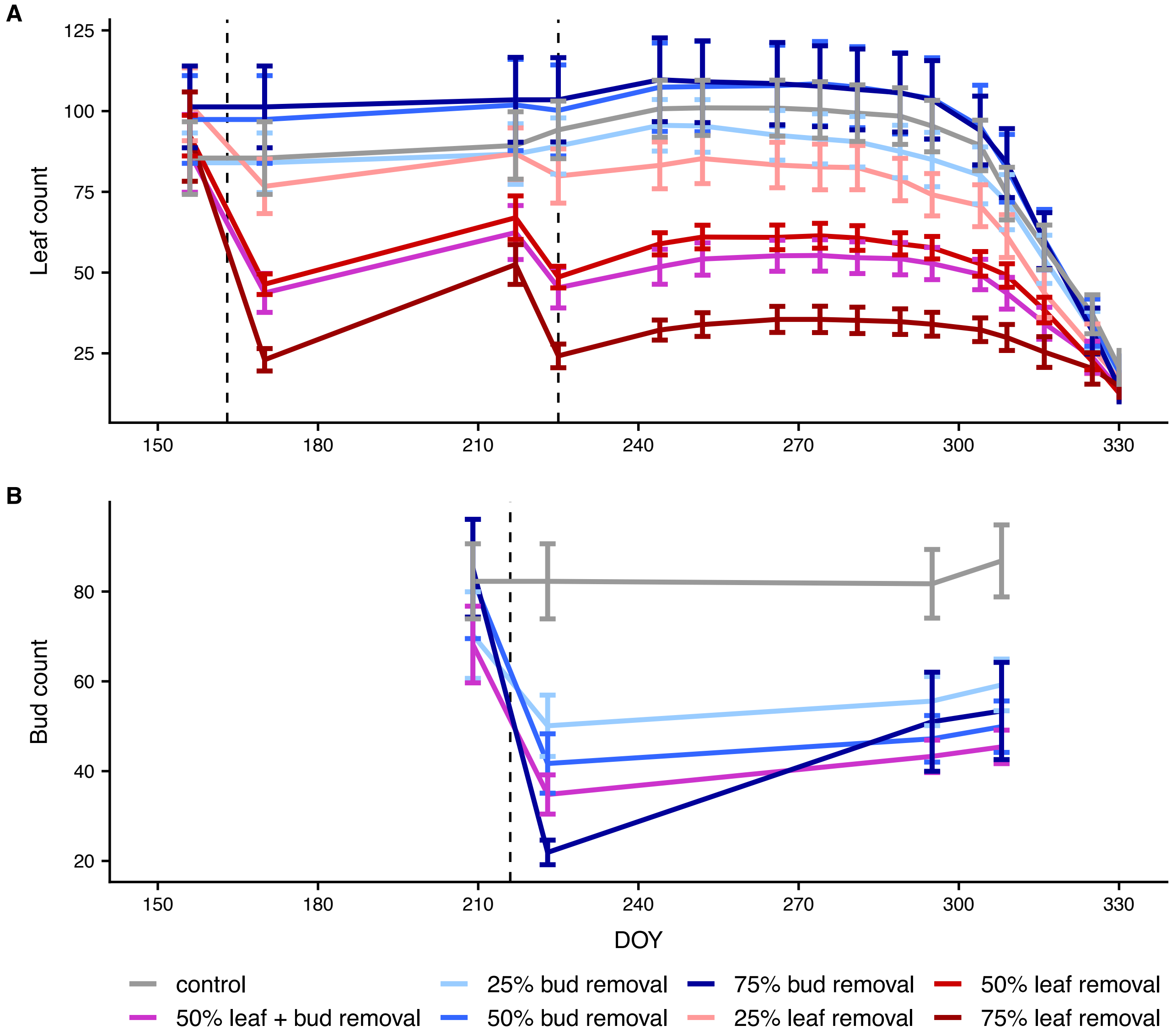

Supplementary Figure 2. Mean absolute leaf and bud counts by treatment over time. The dashed lines mark the DOYs of the leaf **(A)** and bud **(B)** removals. For both leaves and buds, the relative count is defined as the ratio of the current count to the count just before the first respective plant organ removal. For the purpose of visualization, we plotted both the first two leaf and bud count measurements 14 days apart from each other, despite them having happened on the same day right before and after the respective plant organ removal. Measurements done over several days were assigned to the first day of the measurement interval. Error bars mark the area of ± 1 standard error around the mean. A graph of relative leaf and bud counts is depicted in Figure 1.

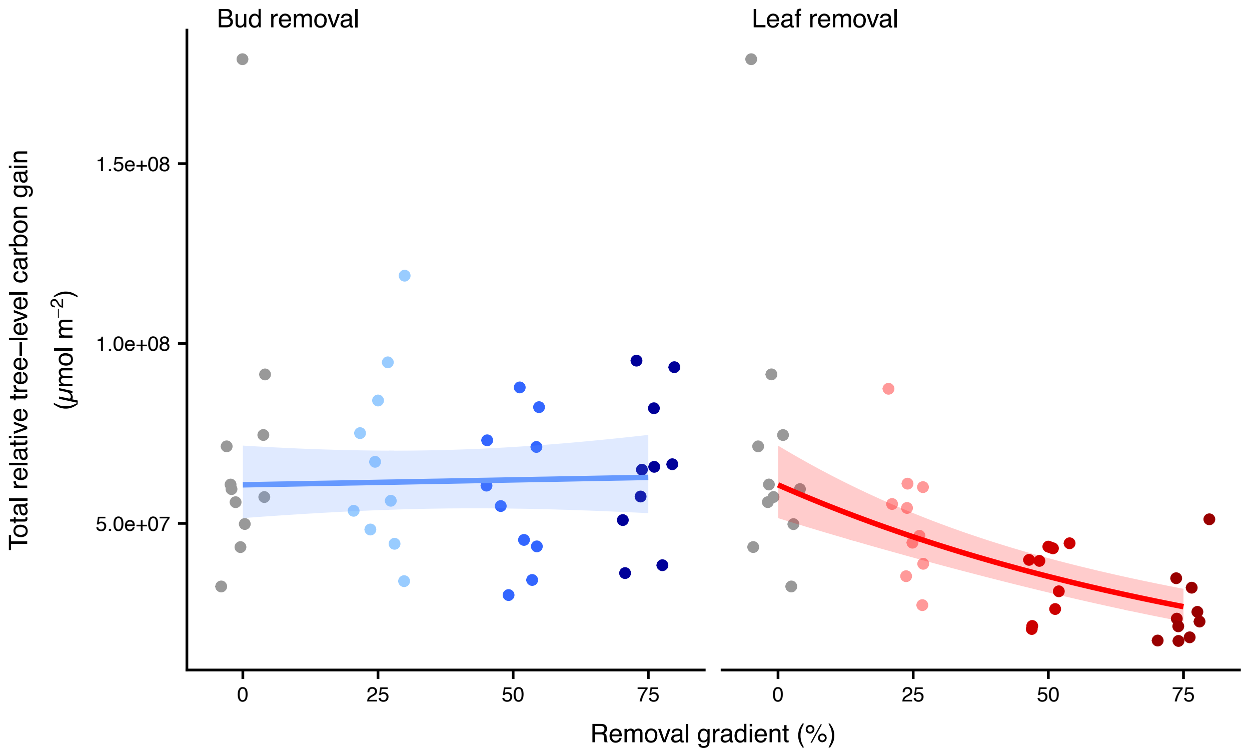

Supplementary Figure 3. Total relative tree-level carbon gain (µmol m^{-2}) across the leaf (right panel) and bud removal gradient (left panel). The colors correspond to the treatment. For each organ removal, we only display the treatments that have 0% of the other organ removed (i.e. the leaf removal panel does only include treatments where bud removal = 0). We added (back transformed) predicted means and approximate 95% confidence intervals for the model of total relative tree-level carbon gain. Model diagnostics are in Supplementary Figure 4. Model results are listed in Supplementary Table 3.

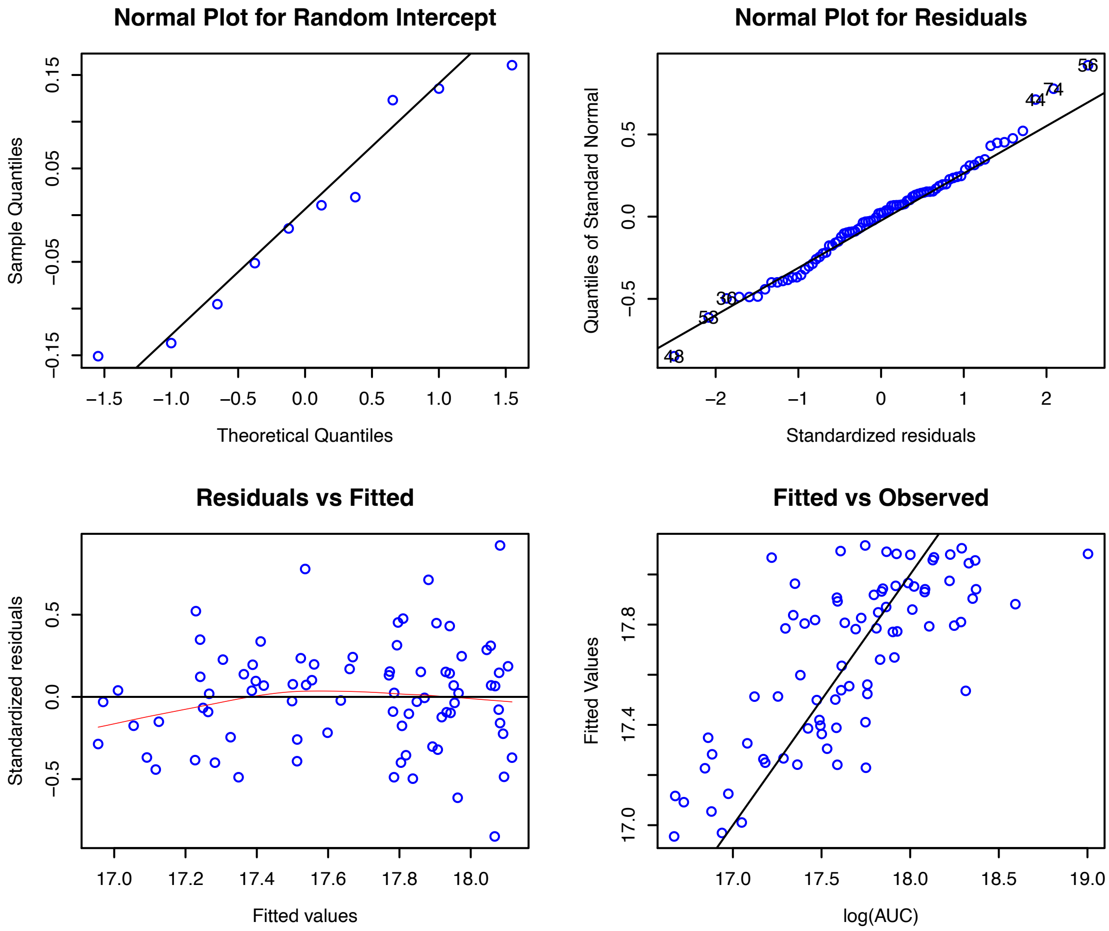

Supplementary Figure 4. Diagnostics plot for the model of total relative tree-level carbon gain (μmol m^{-2}). The data are displayed in Supplementary Figure 3. Model results are listed in Supplementary Table 3.

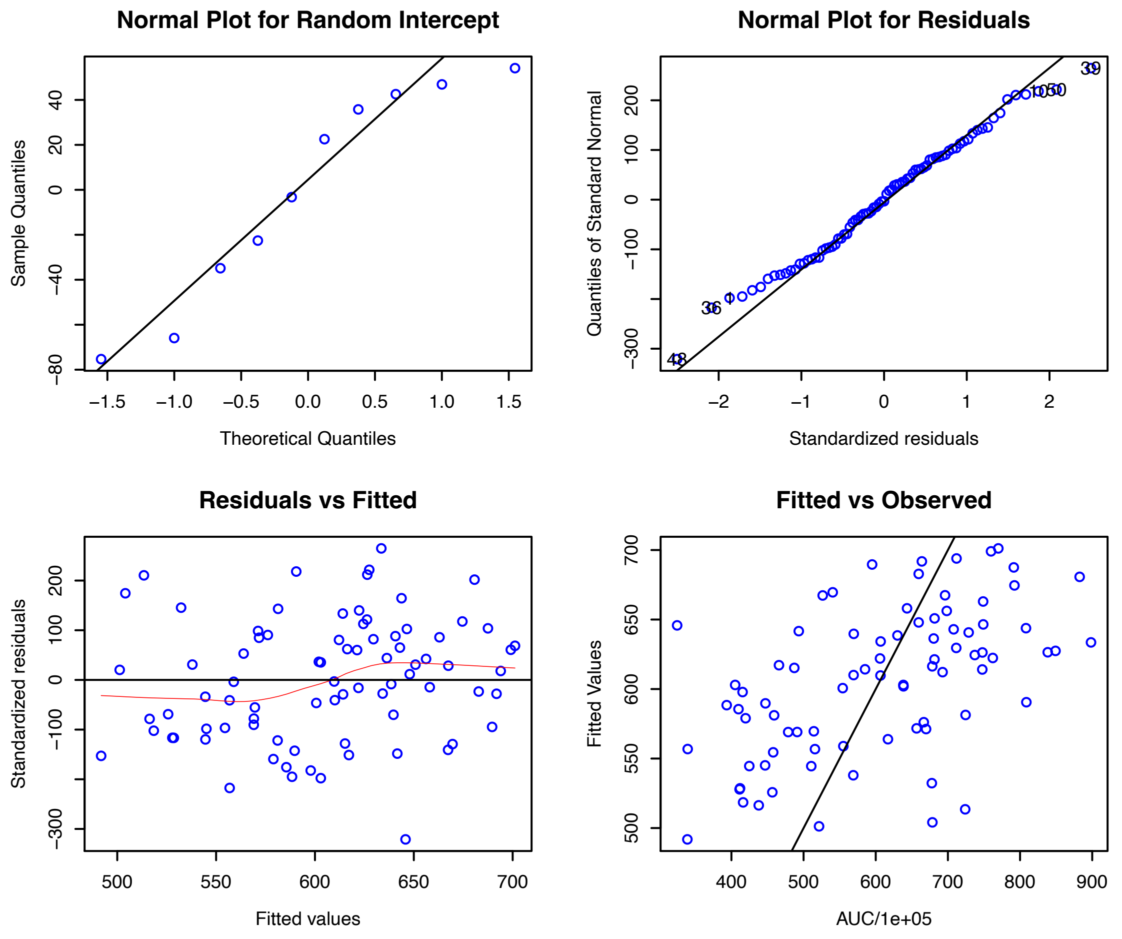

Supplementary Figure 5. Diagnostics plot for the model of the total leaf-level autumn photosynthesis (µmol m^{-2}). The data are displayed in Figure 2. Model results are listed in Supplementary Table 4.

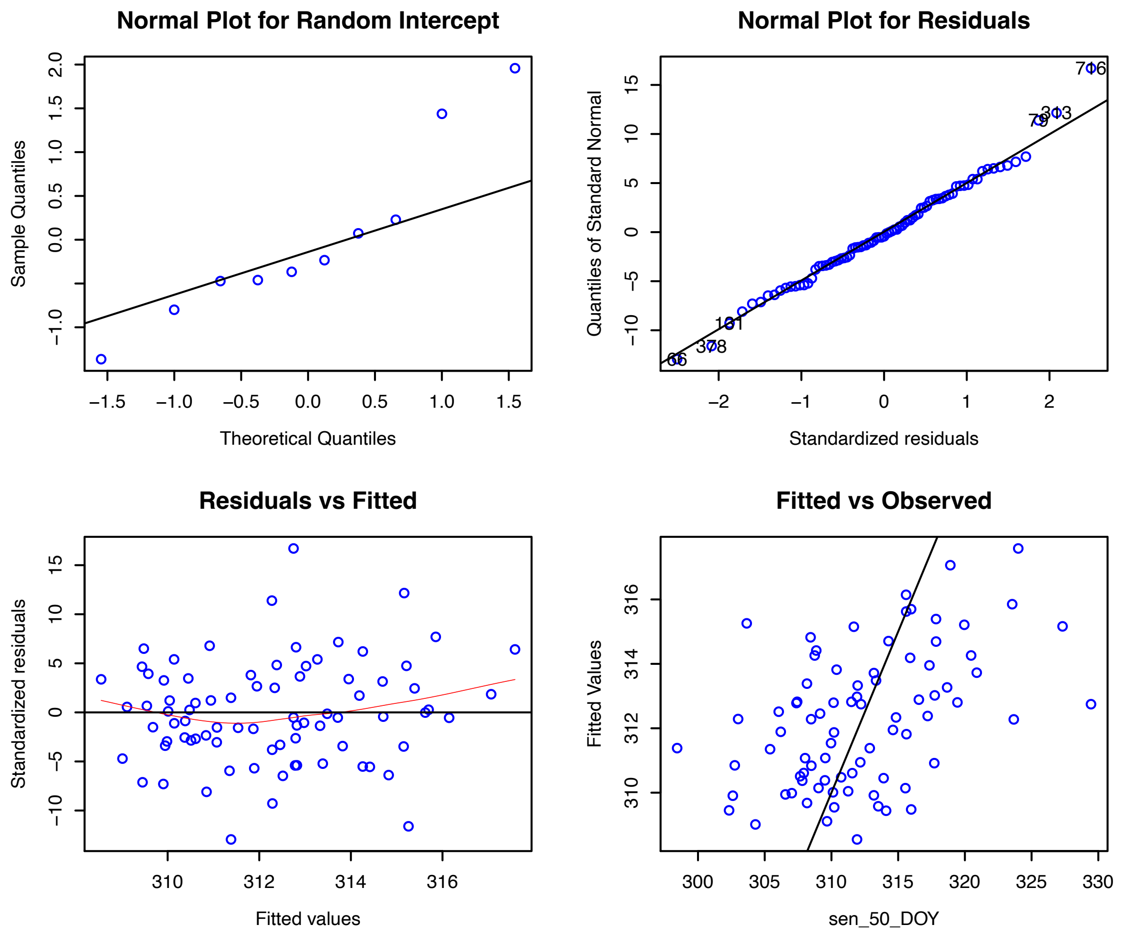

Supplementary Figure 6. Diagnostics plot for the model of the DOY of 50% senescence. The data are displayed in Figure 3 A. Model results are listed in Supplementary Table 5.

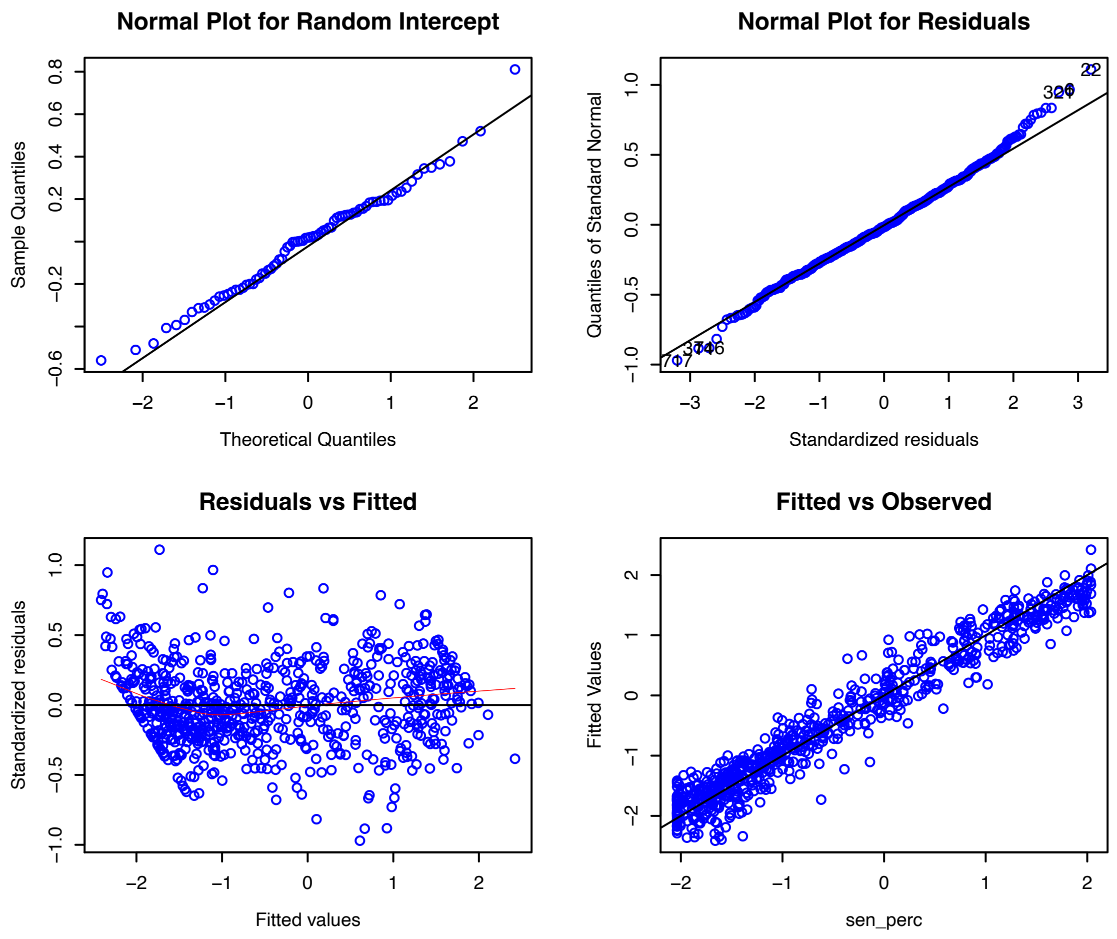

Supplementary Figure 7. Diagnostics plot for the model of senescence over time. The data are displayed in Figure 3 B. Model results are listed in Supplementary Table 6.

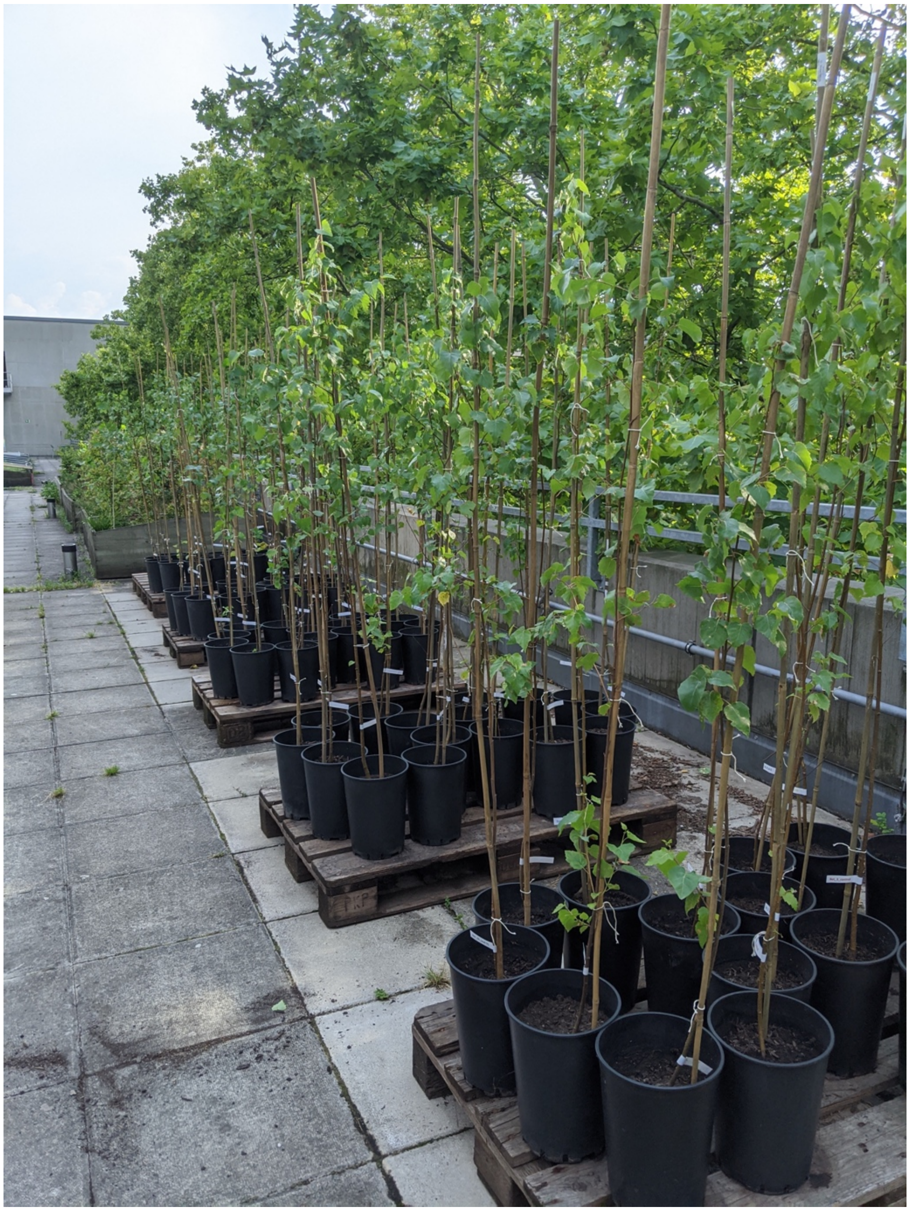

Supplementary Figure 8. Experimental setup on August 10th.

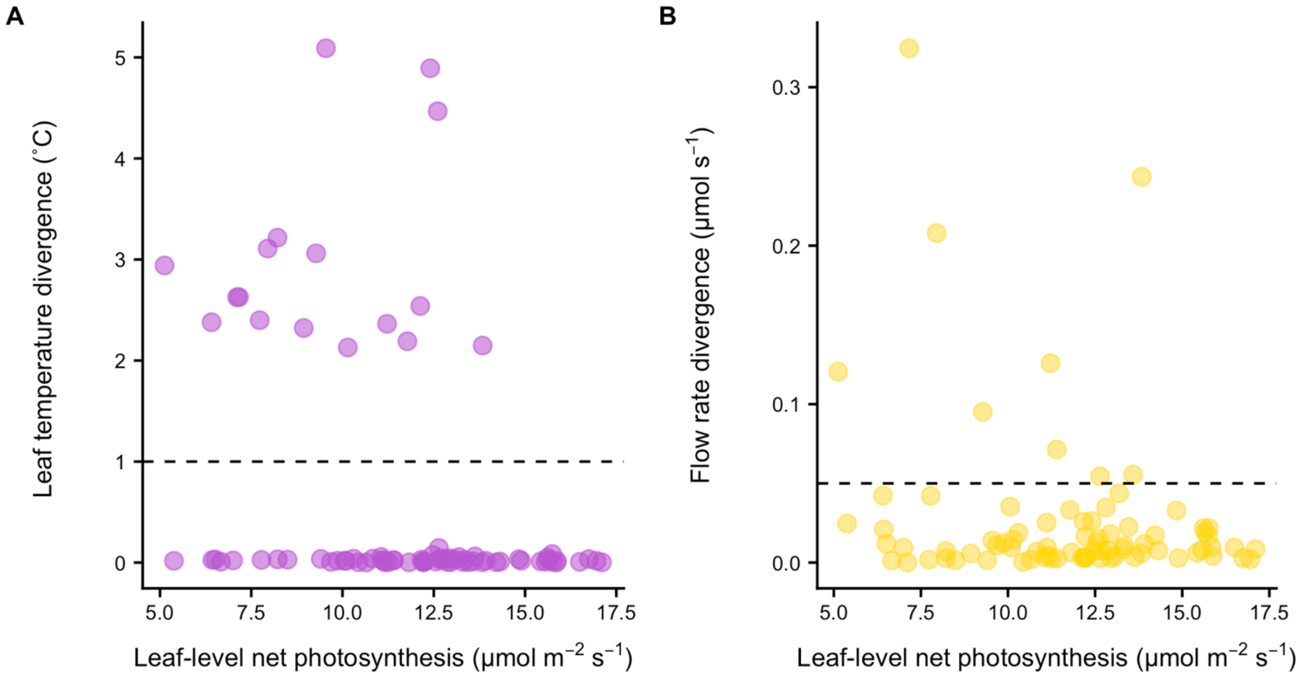

Supplementary Figure 9. Relationship between leaf-level net photosynthesis (µmol m^{-2} s^{-1}) in the first photosynthesis measurement interval and **(A)** the divergence from leaf temperature (˚C) and **(B)** flow rate setpoints (µmol s^{-1}). We chose the first photosynthesis measurement interval for this evaluation because it was the interval where 74% of the leaf temperature–setpoint and 100% of the flow rate–setpoint divergences occurred. The setpoints defined in our LI-COR measurements were a leaf temperature of 25˚C and a flow rate of 500 µmol s^{-1}. The dashed vertical lines indicate what we defined as setpoint divergence, i.e., 0.05 µmol s^{-1} for flow rate and 1˚C for leaf temperature.

Supplementary Tables

Supplementary Table 1. Details for the photosynthesis measurements.

**DOY of sampling event start**	**Date**	**Sampling location**	**Duration of sampling (days)**	**Number of break days**
237	27.08.	Outdoors	5	0
265	21.09.	Outdoors	10	3
281	07.10.	Indoors	4	0
295	21.10.	Indoors	4	0
316	11.11.	Indoors	4	0
330	25.11.	Indoors	3	0

Supplementary Table 2. LI-COR parameter settings.

**LI-COR parameter**	**Setting**
Flow rate	500 µmol s^{-1}
Chamber pressure	0.1 kPa
Relative air humidity	65%
Reference [CO_2]	400 ppm
Fan speed	10,000 rpm
Leaf temperature	20˚C
Light intensity	1000 µmol m^{-2} s^{-1}

Light spectrum	70% red, 30% blue

Supplementary Table 3. Estimated model coefficients for total relative tree-level carbon gain (μmol m^{-2}), which was logged in the model. Significant from zero are marked in bold. df = degrees of freedom, Std. err. = standard error, and R^2 presented as marginal R^2 (conditional R^2). The analysis of variance results for the main effects were: leaf removal (P = 4.754e-14, df = 1) and bud removal (P = 0.757, df = 1). The data and model diagnostics are displayed in Supplementary Figure 3 and Supplementary Figure 4.

**Predictor**	**Estimate**	**Std. error**	**df**	***P***	**R^2**
Intercept	17.92	0.08	32.37	**< 2e-16**	0.42 (0.51)
Leaf removal	-0.01	1.44e-3	69.22	**1.37e-10**	
Bud removal	4.47e-4	1.44e-3	69.22	0.758	

Supplementary Table 4. Estimated model coefficients for total leaf-level autumn photosynthesis (μmol m^{-2}), which was divided by 100,000 in the model. Significant differences from zero are marked in bold. df = degrees of freedom, Std. err. = standard error, and R^2 presented as marginal R^2 (conditional R^2). The analysis of variance results for the main effects were: leaf removal (P = 0.054, df = 1) and bud removal (P = 0.374, df = 1). The data and model diagnostics are displayed in Figure 2 and Supplementary Figure 5.

**Predictor**	**Estimate**	**Std. err.**	**df**	***P***	**R^2**
Intercept	567.17	32.76	29.80	**< 2e-16**	0.04 (0.21)
Leaf removal	1.06	0.55	69.08	0.058	
Bud removal	0.49	0.55	69.08	0.377	

Supplementary Table 5. Estimated model coefficients for the 50% senescence DOY. Significant differences from zero are marked in bold. df = degrees of freedom, Std. err. = standard error, and R^2 presented as marginal R^2 (conditional R^2). The analysis of variance results for the main effects were:

leaf removal (*P* = 0.014, df = 1) and bud removal (*P* = 0.424, df = 1). The data and model diagnostics are displayed in Figure 3 A and Supplementary Figure 6.

**Predictor**	**Estimate**	**Std. err.**	**df**	***P***	**R^2**
Intercept	311.31	1.24	43.82	**< 2e-16**	0.10 (0.17)
Leaf removal	0.06	0.02	69.33	**0.016**	
Bud removal	-0.02	0.02	69.33	0.427	

Supplementary Table 6. Estimated model coefficients for whole-plant leaf senescence over time, which was logit-transformed in the model. Significant differences from zero (for the numeric variables leaf and bud removal) or the DOY 274 (for the factor variable DOY) are marked in bold. df = degrees of freedom, Std. err. = standard error, and R^2 presented as marginal R^2 (conditional R^2). The analysis of variance results for the main effects were: leaf removal (*P* = 0.020, df = 1), bud removal (*P* = 0.697, df = 1), and DOY (*P* = < 2e-16, df = 8). The data and model diagnostics are displayed in Figure 3 B and Supplementary Figure 7.

**Predictor**	**Estimate**	**Std. err.**	**df**	***P***	**R^2**
Intercept	-1.74	0.08	61.06	**< 2e-16**	0.89 (0.94)
Leaf removal	-2.96e-03	1.27e-03	69.30	**0.023**	
Bud removal	4.95e-04	1.27e-03	69.30	0.698	
DOY 281	0.05	4.81e-02	640.00	0.295	
DOY 289	0.44	4.81e-02	640.00	**< 2e-16**	
DOY 295	0.61	4.81e-02	640.00	**< 2e-16**	
DOY 304	1.09	4.81e-02	640.00	**< 2e-16**	
DOY 309	1.63	4.81e-02	640.00	**< 2e-16**	
DOY 316	2.27	4.81e-02	640.00	**< 2e-16**	

DOY 325	3.01	4.81e-02	640.00	**< 2e-16**	
DOY 330	3.32	4.81e-02	640.00	**< 2e-16**	

Supplementary Methods

1. Calculations for second leaf removal

The second leaf removal was needed because there was considerable leaf growth after the first removal and the amount of leaf growth seemed to differ between treatments. In the second leaf removal, we aimed at 1) removing all of the compensatory growth resulting from the treatment as well as 2) removing the portion of the natural leaf growth corresponding to the treatment's specified leaf removal percentage. To determine how many leaves to remove in the second treatment, we did the following calculations:

1. Total leaf growth since 1st leaf removal

For each of the 81 trees in our experiment, we calculated how many leaves had grown in the two months between after the first leaf removal (June 11th) and shortly before the 2nd leaf removal (August 5th) ("absolute total growth"). To account for differences in growth potential between trees of different sizes, we also calculated total growth as the number of leaves grown after the first leaf removal divided by the tree's pre-treatment leaf count before the first leaf removal ("relative total growth").

2. Natural leaf growth since 1st leaf removal

We determined the leaf growth that could be attributed to natural growth, i.e. that is independent of leaf-removal treatments. We calculated one relative natural leaf growth value as the mean change in relative total growth of the 41 trees from non-defoliation treatments. These trees consisted of both the control group as well as all trees from the bud removal treatment, which just had their buds removed on August 3rd–4th. For each of the 81 trees in the experiment, absolute natural leaf growth was calculated by multiplying relative natural leaf growth with each tree's pre-treatment leaf count.

3. Treatment-dependent leaf growth since 1st leaf removal

We considered absolute total growth since the first leaf-removal (step 1) to be made up by two factors, namely absolute natural leaf growth (step 2) and absolute treatment-dependent leaf growth. To estimate the latter, we subtracted each tree's absolute natural leaf growth from its absolute total growth since the first leaf removal.

4. Calculation of leaves to be removed in 2nd leaf removal

For the trees in a leaf-removal treatment, we removed the portion of their absolute natural leaf growth corresponding to the treatment's specified leaf removal percentage (e.g. 25% of absolute natural leaf

growth was removed from trees in the low leaf removal treatment) as well as all leaves resulting from treatment-dependent growth.

We assumed that natural leaf growth happens before treatment-dependent leaf growth, i.e. all leaves were assigned to absolute natural leaf growth first and only once this number was reached, we considered leaves to be a result of treatment-dependent growth. Accordingly, for all trees who had a negative absolute treatment-dependent leaf growth value because their absolute total leaf growth was less than what we expected to be their absolute natural leaf growth, we assumed that all grown leaves were due to natural growth and no treatment-dependent growth had occurred. In each case, the total number of leaves removed was capped by the tree's absolute total leaf growth.

2. Trustworthiness checks of photosynthesis measurements

We checked the trustworthiness of each leaf-level net photosynthesis value by applying criteria on the biological meaningfulness of the additional data in the LI-COR log file. We excluded observations that had negative stomatal conductance or transpiration values because such values are not biologically possible. Further, we demanded that leaf temperature did not equal air temperature in the measurement chamber as this indicated that there had not been contact between the thermocouple of the LI-COR and the leaf. All of the observations in the dataset passed these filters. Finally, we evaluated the two observations where the absolute difference between sample and reference [CO_2] was below the instrument precision (0.1 μmol mol^{-1}). Since these two values were obtained in the last measurement interval, we assumed that such low photosynthetic rates were reasonable and we kept them in the dataset.

There were additional criteria we explored. As transpiring leaves are expected to have a lower temperature than the air, it seemed reasonable to consider filtering out observations where leaf temperature was higher than air temperature in the chamber. In our case, 95% of the LI-COR photosynthesis measurements had higher leaf than air temperatures. However, we assume that this is owed to the low transpiration values of our plants (average: 0.001 mol m^{-2} s^{-1}) and not due to flawed measurements, which is why we did not apply this filtering criterion.

We also investigated how much flow rates and leaf temperatures diverged from their respective setpoints (500 μmol s^{-1}, 25˚C). Of the 478 observations in our final dataset, 9 observations diverged more than 0.05 μmol s^{-1} from the flow rate setpoint and 23 diverged more than 1˚C from the temperature setpoint. However, as the leaf-level net photosynthetic rates of the diverging observations were within the biologically reasonable intervals defined by the rest of the data (Supplementary Figure 9), we kept all observations with flow rates and leaf temperatures diverging from their respective setpoints.

3. Dataset manipulations for modeling

To satisfy model assumptions, we transformed the data where needed.

The inverted relative SPAD index was logit-transformed as follows:

$$\text{inverted rel. SPAD index}_{\text{transf.}} = log\left(\frac{(inverted\ rel.SPAD\ index + 0.15)}{(1 - inverted\ rel.SPAD\ index + 0.15)}\right) \quad \text{(S1)}$$

The summand 0.15 in Equation S1 was chosen through visual inspection of the curve.

The total relative tree-level carbon gain was log-transformed as follows:

$$\text{total rel. tree-level carbon gain}_{\text{transf.}} = \log(total\ rel.\ tree\text{-}level\ carbon\ gain) \quad \text{(S2)}$$

Total leaf-level autumn photosynthesis was divided by 100,000.
